# 

**Published:** 1910-10-08

**Authors:** 


### CONTENTS.

| | PAGE |
|---|---|
| **The Spread of Poliomyelitis in America** ... ... | 35 |
| **Inquests after Operations** ... ... ... ... ... ... ... | 36 |
| **Annotations—** | |
| The Committee on Colour Blindness—Trachoma in Queensland—Osteopathy ... ... ... ... ... ... ... ... | 37 |
| **Medical Opinion and Movement** ... ... ... ... ... | 38 |
| **Hospital Clinics—** | |
| Inflammation of the Iris. By A. Maitland Ramsay, M.D., F.R.F.P.S.G. ... ... ... ... ... ... ... ... ... | 41 |
| **Special Article—** | |
| The Hebrew Physicians of Mediæval Avignon ... ... ... | 43 |
| **Practical Notes on Diagnosis and Treatment** ... | 44 |
| **Surgery—** | |
| The Importance of Examining the Teeth ... ... ... ... | 45 |
| **Dermatology—** | |
| The Microbacillus of Acne ... ... ... ... ... ... ... | 46 |
| Tinea Cruris ... ... ... ... ... ... ... ... ... ... ... | 46 |
| **Medico-Legal Points—** | |
| Identity of the Living and Dead ... ... ... ... ... | 47 |
| **News and Events of the Week** ... ... ... ... ... | 49 |
| **The Week's Work—** | |
| The King Edward Memorial Proposals—The Glasgow Conference—The Medical Exhibition—Hostels in the Country—The Green Lady Hostel—This Week's Drug Market ... ... | 51 |
| **Parliament and Publications—** | |
| Death-rate Among Seamen ... ... ... ... ... ... ... ... | 52 |
| **Special Institutional Articles—** | |
| The British Hospitals Association ... ... ... ... ... ... | 53 |
| The Legal Relations of Hospital, Hospital Staff, and Patient ... | 55 |
| The Croydon Cottage Hospital for Felixstowe and Walton ... | 57 |
| Scottish Asylums ... ... ... ... ... ... ... ... ... ... | 58 |
| **Institutional Notes and News** ... ... ... ... ... ... | 59 |
| **The Hospital World** ... ... ... ... ... ... ... ... ... | 60 |
| **Gifts and Bequests** ... ... ... ... ... ... ... ... ... | 61 |
| **Jottings** ... ... ... ... ... ... ... ... ... ... ... ... | 61 |
| **Editor's Letter-Box—** | |
| The Green Lady Hostel—Osteopathy ... ... ... ... ... | 62 |

